# Intra-substance meniscal changes and their clinical significance: a meta-analysis

**DOI:** 10.1038/s41598-021-83181-5

**Published:** 2021-02-11

**Authors:** Rani Ahmad

**Affiliations:** King Abdulaziz University, Jeddah, Saudi Arabia

**Keywords:** Health care, Medical research

## Abstract

The degeneration of radial tie fibres of the central meniscal layer, and thinning of its lamellar layer results in increased intensity signals on magnetic resonance imaging, making it difficult to differentiate from true meniscal tear. This study aimed to assess the rate of encountered MRI grades 1 and 2 intrasubstance meniscal changes, and to set guidelines to report these changes based on predicted clinical outcome. A systematic review approach was employed using search engines, libraries, and databases (Google Scholar, ERIC, PubMed, and Medline) to search for scholarly sources on meniscal lesions and their significance in MRI published between 1 January 2000 and 30 June 2019. It retrieved 2750 abstracts, out of which 2738 were excluded and 13 studies meeting inclusion criteria were meta-analysed. It found an association between intrasubstances meniscal changes and outcomes. It resulted that intrasubstance meniscal changes were preservable through the protective functioning of the meniscus. Other than weight gain, no other significant risk factor of developing true meniscal tears later in life was found. It is important to examine intrasubstance meniscal change when patients suffer from mechanical meniscal symptoms especially in old age.

## Introduction

Magnetic resonance imaging (MRI) is a common radiological technique used to diagnose or examine the knee joint. There are various causative factors of knee pain, such as trauma or degeneration, which can directly affect the meniscus^1,2^. The meniscus plays an important role in nitrifying articular cartilage, load transmission, lubrication, and shock absorption. The wedge-shape of the meniscus renders multiple and complex functions by stabilizing the femoral condyle in its articulation with the flat surface of the tibial plateau^3^. This structure enables the conversion of vertical compressive forces into horizontal hoop stress. However, for some experts, the meniscus is often considered an unimportant vestigial structure, with no evident function^4^.

Several forces have been identified that cause or lead to meniscal tears, such as compression and shear tension. Meniscal deformation is likely to take place when shear forces develop between the internal collagen fibres^3^. Also, direct trauma may lead to meniscal tear after the degeneration of the meniscus. In cases where a patient is overweight, the knee can undergo degeneration, especially when the patient does not engage in healthy movement of the knee^5^. Multiple repeated microtraumas can lead to intrasubstance degeneration of the meniscus^6^. A meniscal tear can occur alone or in an association with other proximal tears, such as the anterior cruciate ligament (ACL) and collateral ligaments. Discoid meniscus, where the crescent-shaped meniscus is thickened, is a rare congenital anomaly but presents a higher risk of developing a tear^7^. Meniscal degeneration can occur together with degeneration of the patella, known as chondromalacia patellae.

The detection and evaluation of meniscal changes are made using MRI, which utilizes multiple sequences in various planes. The T2-weighted and PD-weighted sequences are the most commonly used in coronal and sagittal planes. These sequences are highly sensitive to myxoid degeneration and edema, which can occur during meniscal tears. Therefore, MRI is broadly considered a highly accurate imaging method to diagnose meniscal tears^8^.

Normal meniscus for the adult patient usually appears as a low signal intensity structure in T2 and PD imaging sequences, although subtle high signal intensity may be found within the substance of the meniscus. The subtle increase in signal intensity contributes to the normal content of the meniscus as well as the existence of meniscal vessels that is easily observed among young patients^9^.

The morphology of the meniscus, including its surface structure, is one of the differentiating points between true meniscal tear and degeneration^10^. Based on Beals et al., meniscal changes are graded into three categories^10^.

**Figure 1:**
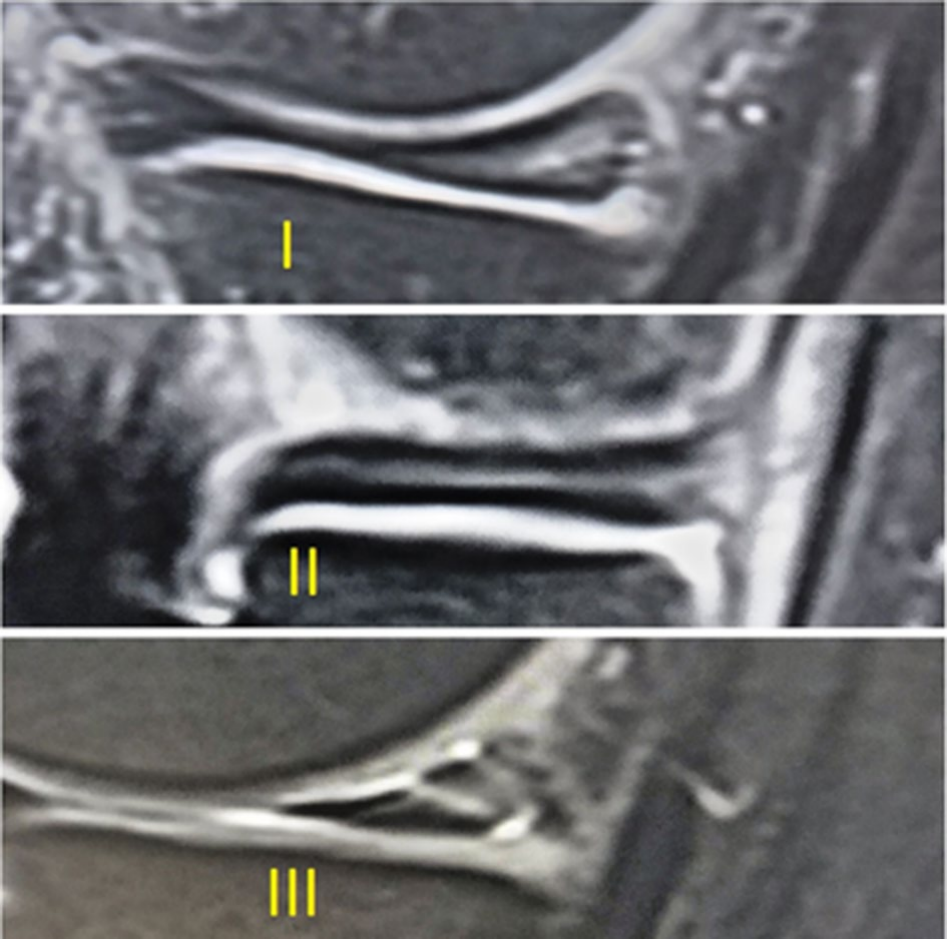
Meniscal changes grading scale on MRI.

- Grade 1 changes appear as the rounded increased signal intensity does not reach any of the meniscal surfaces.
- Grade 2 changes are demonstrated as linear increased high signal intensity does not reach the meniscal surfaces.
- Grade 3 meniscal changes reach to at least one of the articular surfaces and are usually easily recognized. They represent true meniscal tears that can be categorized depending on direction, complexity, and the presence of displacement (Fig. 1).

The increased intensity of MRI signals of grade 1 or 2 are the result of degeneration of radial tie fibers of the central meniscal layer, and thinning of the lamellar layer^11^. The differentiation between grades 1, 2, and 3 is important to provide appropriate diagnosis and treatment, and leads to positive healthcare outcomes. The present study aims to evaluate intrasubstance meniscal changes and their clinical significance. We expect to find grade 1 objectives also include to find grade 1 and 2 intrasubstance meniscal changes encountered toward guidelines for reporting changes appropriate to predict clinical consequences. The findings of this study may contribute to clinical practice of reporting change even for asymptomatic patients.

## Methods

The study methods were based on the guidelines set out in the Preferred Reporting Items for Systematic Reviews and Meta-Analyses (PRISMA) program.

### Search strategy and study selection

**Figure 2:**
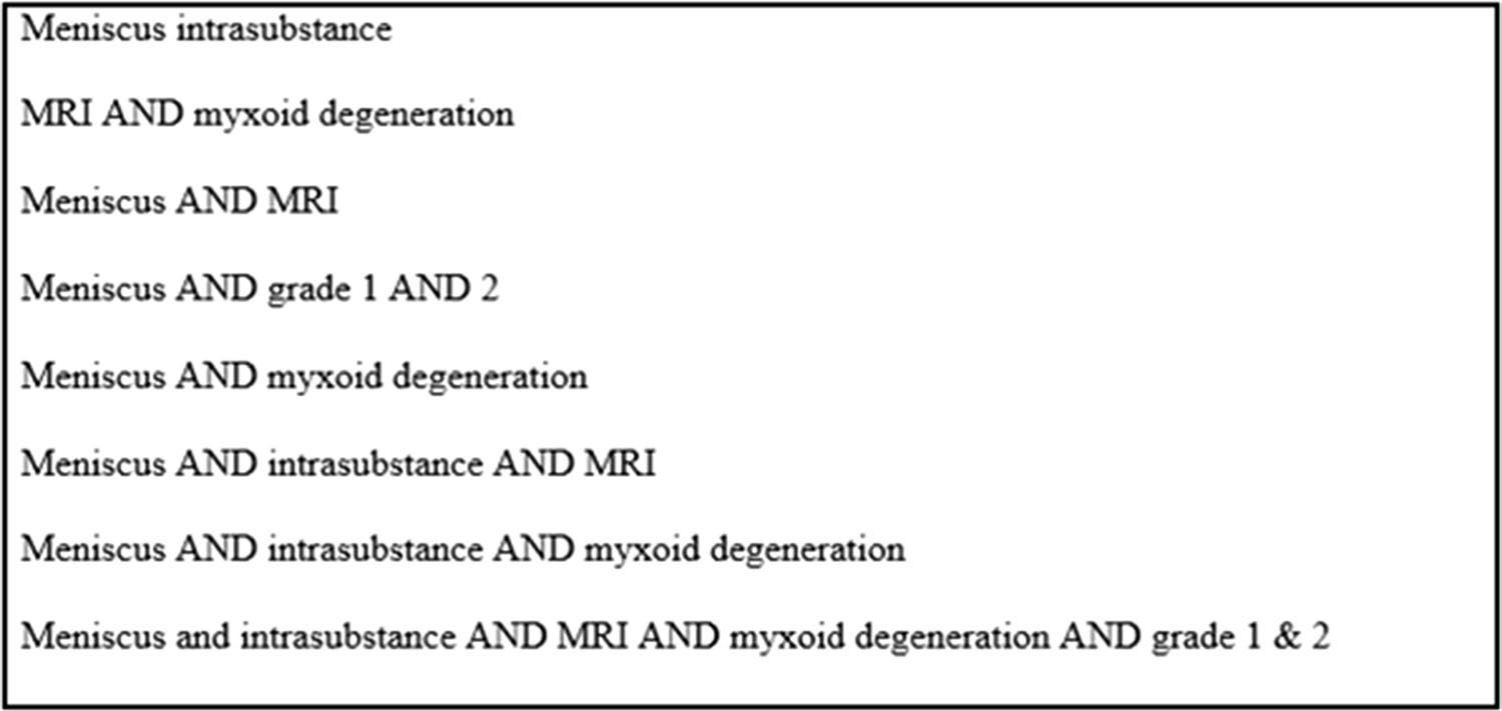
Search strategy (keywords).

The present study has followed a broad spectrum of research tools, including libraries and scholarly platforms such as Medline, ERIC, PubMed, and Google Scholar in order to cover a wide range of studies. Studies on the significance of intrasubstance meniscal lesions on MRI were included for systematic analysis. The databases searched to find relevant literature were; Medline, PubMed, Web of Knowledge, CINAHIL, and Delphi. The main keywords used in the search are: ‘meniscus OR intrasubstance’, ‘MRI AND myxoid degeneration’ and ‘myxoid degeneration AND grade 1 & 2’ (where ‘OR’ and ‘AND’ represents binary operators). The sequence of searched keywords is shown in Fig. 2.

The search was limited to studies published in English between 1 January 2000 and 30 June 2019. The reference lists of articles considered important in this stage of the study were included by the researchers.

The review process was regulated in a two-staged process. In the first phase, each study was evaluated by the investigators along with an academic professional. Endnote library was created to list all the screened searches for full-text eligibility screening. The studies were included for further analysis if they were based on retrospective, prospective, or cross-sectional analysis. In the second phase, the extraction of articles took place, where studies were reviewed based on titles and abstracts. Studies relevant to the topic were extracted until the selection of a desirable number of articles.

### Eligibility criteria

Research articles such as, observational and clinical studies, cross sectional, cohort studies, and case studies were considered; all other literature categories were excluded. Only studies published in 2000–2019 were considered. Older versions of studies, essays, and blogs were excluded because of reduced reliability. The study adjusted the search strategies based on the specifications of the individual search engines and databases. No search restrictions were predefined for follow-up time, age, or study size. Reference lists of the included studies were reviewed for identifying additional studies. Disagreements on the inclusion of studies were resolved by mutual agreement among reviewers.

### Data extraction

Using descriptive table, characteristics of the study, such as first author, year of publication, sample size, study design, primary outcome and level of evidence were extracted. Uncovering the rate of intrasubstance meniscal changes in MRI relating to the pre-specified graded criteria was considered the primary outcome. A published hierarchy of patient-reported outcome measures was followed including the constructs for measuring their reliability, validity, and responsiveness. Outcomes concerned with intrasubstance meniscal changes for all reported assessments were extracted from the included studies. Standard deviations, confidence intervals, and *P* values, and interquartile ranges, were extracted from the data of the studies. If required, means and measures of dispersion from figures in the included studies have been approximated or rounded. The presence or absence of intrasubstance changes were expressed categorically (e.g., N(%) or odds ratios) and with *P* values.

### Synthesis of results

The study calculated the effect sizes in the individual studies as standardized mean differences for the analysis of meniscal changes, which allow pooling and comparison of the different outcomes across trials. However, using standardized mean difference, the estimate of the effect size is presented with a slight bias that overestimates the effect size.

Chi-squared test for the homogeneity and Cochran’s Q of studies were analysed for heterogeneity of data. As the heterogeneity of the data was low, the studies were included in the meta-analysis. For meta-analysis, data was analysed and summarized relating to intrasubstance meniscal lesions using the Stata software package, version 13.0. Standard errors and odds ratios were extracted from the published papers. The odd ratios were measured to identify the influence of age on the development of meniscal changes. Similarly, effect measures, age groups, and studies on the general population were used to pool the studies. Random effects models of the odds of morbidity and the subsequent 95% confidence intervals were obtained for summary estimates.

### Risk of bias assessment

Based on the guidelines described in the *Cochrane Handbook for Systematic Reviews of Interventions*^12^, two reviewers independently evaluated the risk of bias. The two reviewers evaluated sequence generation, blinding, handling of incomplete outcome data, allocation concealment, selective outcome reporting, and other biases in the studies on meniscal changes. Each of the domains was scored as unclear, inadequate, or adequate. Ambiguities were resolved by consensus. To avoid the possibility of bias, a third reviewer was contacted to provide assistance.

## Results

**Figure 3:**
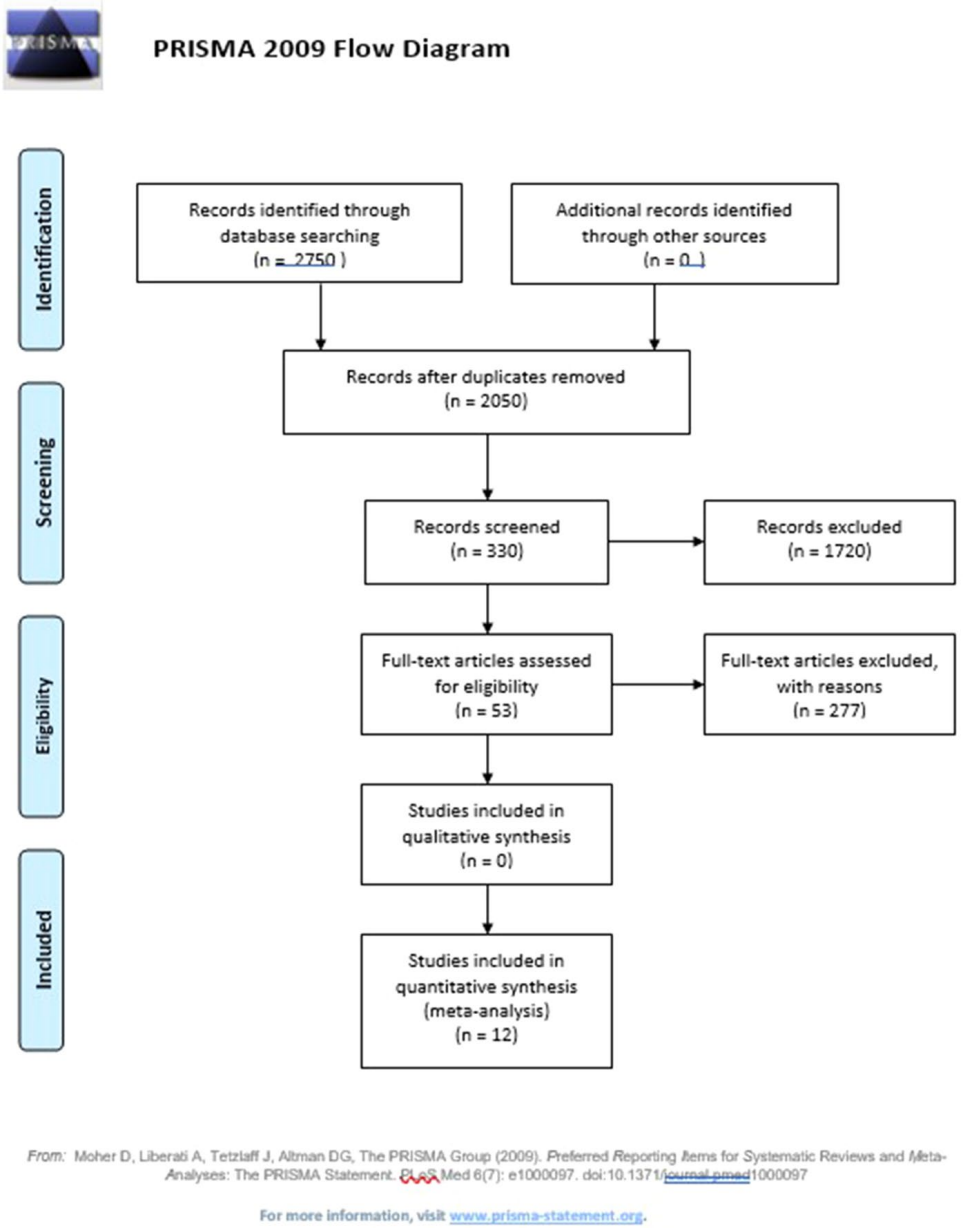
Prisma flowchart.

The literature search resulted in the retrieval of 2750 abstracts based on the study keywords and the inclusion and exclusion criteria. Abstracts discussing the significance of intrasubstance meniscal lesions on MRI were selected. This first phase resulted in the exclusion of 88% of the abstracts (approximately 2420 abstracts). The remaining 330 articles were carried over to the second phase and 53 articles were retrieved for further analysis resulting in the final selection of 13 articles for systematic review analysis. The entire search process is shown in PRISMA flowchart in Fig. 3.

The risk of bias and the quality of the included studies were evaluated through Cochrane tool^13^. The development of the review and the preparation of the meta-analysis were followed using the PRISMA guidelines.

The meta-analysis was conducted following the inclusion of 13 articles with the implementation of the inclusion criteria. Table 1 provides the compiled results obtained from the studies that show the association between the intrasubstance meniscal changes and provides a detailed classification of the studies included.

**Table 1:** Characteristics of included studies.

Name of author	Year of publication	Study type	Study methods and sample size	Final outcomes	Level of evidence
Li et al. ^11^	2013	Prospective cohort study	Correlation between MRI and histology was obtained from 21 patients in a prospective manner. Proton density weighed turbo spin echo MR images were used	Findings identified that high signal intensity on MRI is caused through minimal layer thinning of the lamellar layer, radial tie fibers, and degeneration of the central layer of the meniscus	Moderate
Guimaraes et al. ^14^	2018	Prospective cohort study	487 subjects were studied from baseline values, and after 48 months, by using 3-T MRI. Participants included those with weight loss, weight gain, stable weight, and substantial weight gain. Meniscal intrasubstance degeneration was observed through WORMS grading system	It has been shown that progression from meniscal intrasubstance degeneration to meniscal tear or maceration was caused by weight gain. Moreover, A significant 5-fold increase was observed in people with normal weight, while in other conditions of severe weight gain, a tenfold increase in progression was observed	Moderate
Crema et al. ^15^	2010	Prospective cohort study	The study has performed cartilage segmentation in different tibiofemoral subregions, using computer software	Findings indicated a significant association between cartilage loss and true surface meniscal tears, whereas no significant change in terms of increased cartilage loss was observed	Moderate
Kumm et al. ^16^	2015	Prospective cohort study	269 patients aged 45–55 years without knee osteoarthritis and medial meniscal tear were studied. 3 T MR images were assessed at the baseline of 24, 48, and 72-months follow-up. The risks of medial meniscal tear, with respect to age, sex, BMI, and knee side were evaluated through log–log model	Findings of the study indicated that linear intrameniscal signal intensity was observed among 140 patients, specifically in the medial compartment. In patients without OA, MRI linear intrameniscal signal intensity failed resolve over 6-year follow up and is considered as the risk factor for medial degenerative meniscal tears	High
Englund et al. ^17^	2008	Prospective Cohort study	The study recruited 991 subjects to assess the integrity of menisci in the MRI scan. Questionnaire was used to evaluate the presence of any mechanical meniscal symptoms	The prevalence of meniscal tears was common among both female and male patients. However, incidence level was higher among older patients, and also specifically for females belonging to the age group between 70 and 90 years	Moderate
Yoo et al. ^18^	2012	Retrospective cohort study	MRI scans and intraoperative videos of children with discoid lateral meniscus (DLM) were reviewed. MR images of DLM were graded for the sample of 79 children. Signal and morphological changes associated with DLM on MRI were then correlated	Findings of the study showed that most of the patients had meniscal changes of grade 1 and grade 2, showing a dot-like intrameniscal signal change and band-like intrameniscal change. However, no accurate prediction of grade 3 is possible without the identification of grade 3 tears	High
Hagino et al.^19^	2011	Retrospective cohort study	Included 8 patients were with stable knee and no ligament injury, and had only isolated anterior horn tear of the lateral meniscus	The mean lysholm score was 65 before surgery and recovered to 89 at the last follow-up, on average 12 months after surgery	Moderate
Sproule et al. ^20^	2005	Retrospective cohort study	A total of 68 patients with a mean age of 28 years were included. These patients were suspected with post traumatic internal derangement of the knee, and had MRI prior to arthroscopy	According to the findings, out of 68 patients, equivocal meniscal changes of grade 2 were detected among 10 patients. However, it was concluded that meniscal tear was unlikely to appear when focused with high signal intensity. It was further suggested to consider patient history and past clinical examination before commencing with MRI diagnosis at altered signal intensity	Moderate
Guimaraes et al. ^21^	2017	Case–control study	57 subjects with an ACL tear were included for screening of the intrameniscal signal intensity alteration. Before ACL reconstruction, compositional and morphological MRI was performed, while clinical outcomes were measured using the KOOS scale	MRI findings of intrameniscal tears are common among patients with ACL tears. However, changes of the benign course were visible within the follow-up period of 24 months. Also, no change of KOOS values were identified before and after 24 months among patients with and without intrameniscal signal intensity alteration	High
Saseendran et al. ^22^	2018	Cross sectional study	A total of 269 patients without medial meniscal tear at baseline were studied	The presence of linear intrameniscal signal intensity on MRI is a major risk factor for medial degenerative meniscal tear	Moderate
Low et al. ^23^	2008	Pilot study	10 patients with a mean age of 28 years presented to the senior orthopaedic surgeon in a duration of one year were included, with a report of grade 2 meniscal changes and suspected meniscal tear	All the patients had grade 2 meniscal changes in their report. Arthroscopy resulted in the detection of intrasubstance tears among all patients. The study concluded that in cases where grade 2 changes on MRI correlates with the site of symptoms, arthroscopic examination should be preferred for the visibility of intrasubstance meniscal tear	Moderate
Nguyen et al. ^24^	2014	Literature review study	It is a literature review-based study that has used secondary sources to obtain results	Important distinction indicated between horizontal tear and intrasubstance changes. Studies suggested the use of two slice touch for the visibility of a true tear. Findings further indicated that intrasubstance changes are at minimum risk of developing tear. However, meniscal tears are further associated to the degenerative knee	Low
Howell et al. ^25^	2014	Review article	The degenerative process of meniscus, along with different diagnostic and treatment modalities were reviewed	Both surgical and non-surgical methods are effective. However, more studies are recommended to study the long-term effect of these treatment modalities	Moderate

### Risk of bias

**Figure 4:**
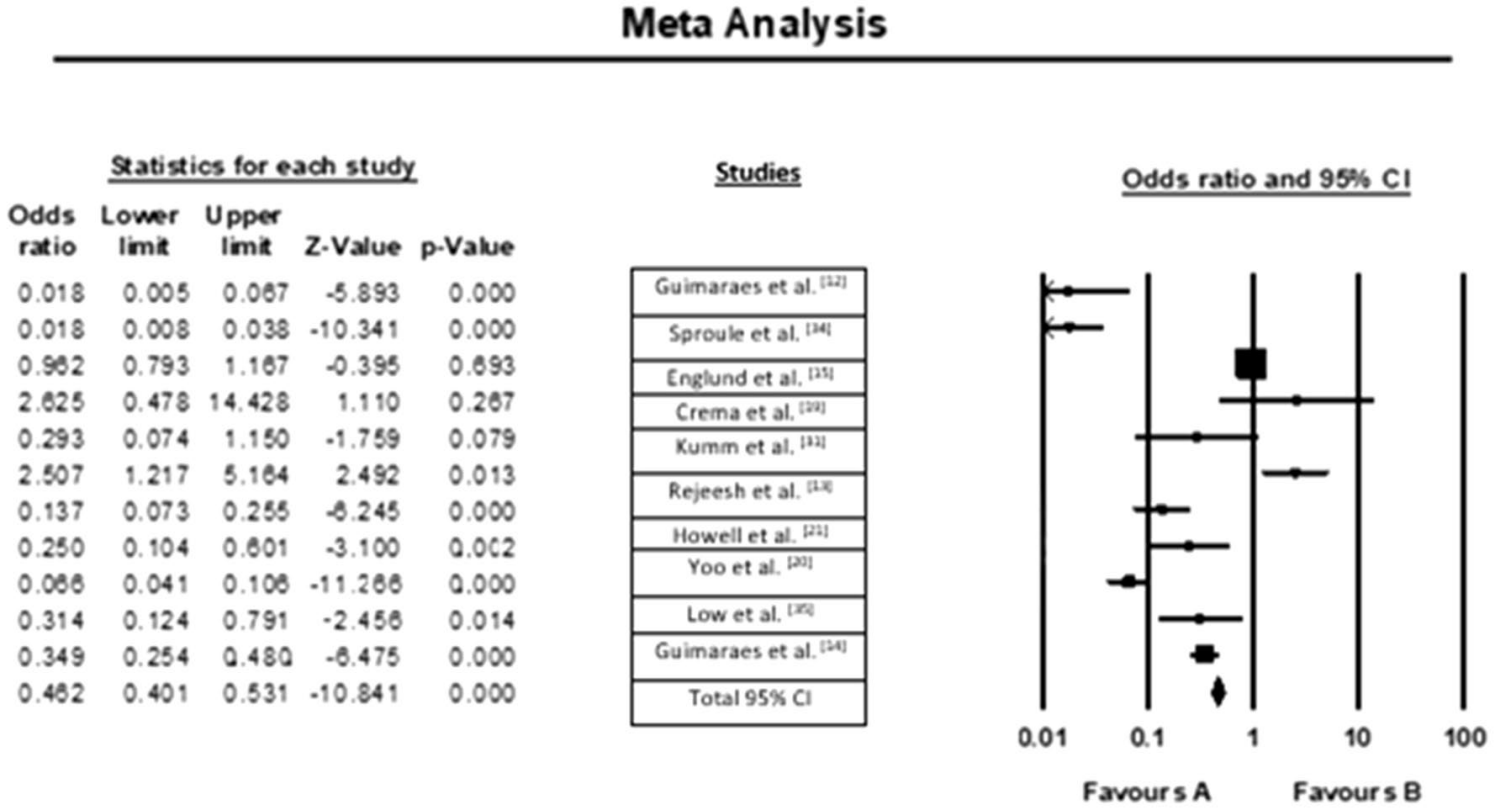
Risk of bias summary.

Agreement between evaluators on the risk of bias varied from 80 to 100%. Two reports were considered as adequate on all domains, while three reports were evaluated for blinding to minimize bias and maximize the study validity. The remaining studies were not blinded (Fig. 4).

### Study characteristics

Table 1 shows that the majority of the studies were prospective cohort studies^11,14–17^, whereas, three were retrospective cohort studies^18–20^, others were case–control^21^, cross-sectional^22^, pilot^23^ and review-based studies^24,25^.

The 13 studies recruited were based on the absence of actual tear representing the intrameniscal changes and other clinical features. Different treatment modalities were observed, including surgery and intra-articular injection, and conservative treatment, such as physiotherapy, weight modification, and oral medicines, which are likely help in improving the symptoms. Table 1 presents the aims, methods applied, and final outcomes of the included studies concerned with intrasubstance meniscal changes.

Table 2 presents studies reporting meniscal changes among patients with MRI diagnosis in terms of mean (M), standard deviation (SD) and P-values associated with meniscal changes. The mean and standard deviation values are provided in relation to the age of the participants. The results show that most studies report significant meniscal change with respect to age group. For instance, in the study of Saseendram et al.^22^, patients with a mean age of 35 years showed significant meniscal changes. This is similar to a number of other studies, i.e., Englund et al.^17^, Nguyen et al.^24^, Guimaraes et al.^21^, and Kumm et al.^16^, which found significant meniscal changes in patients with the mean age of 35, 48, and 41 years. This indicates that an increase in age influences the development of meniscal change.

**Table 2:** Studies reporting meniscal changes among patients on MRI.

Publication	Meniscal changes reported among patients on MRI based on their age group	
Guimaraes et al. ^14^	M = 39.5, SD ± 1.3	< 0.001
Saseendram et al. ^22^	M = 35.2, SD ± 2.5	> 0.001
Englund et al. ^17^	M = 35.1, SD ± 3.1	> 0.001
Sproul et al. ^20^	M = 32.028, SD ± 2.2 ± 1.4	< 0.001
Hagino et al.^19^	M = 32.0, SD ± 2.2	> 0.001
Nguyen et al. ^24^	M = 41.2, SD ± 1.9	> 0.001
Guimaraes et al. ^21^	M = 35.6, SD ± 1.5	> 0.001
Kumm et al. ^16^	M = 48.3 SD ± 3.1	> 0.001

Table 3 shows the age-related risks of meniscal changes among patients in individual studies. The odd ratios in the table indicate the possibility to which patients’ age affect the development of meniscal changes. The highest odds ratio, i.e., 74.3, was attained for the study of Englund et al.^17^, which consisted of patients in the age group 49–61 years, while the lowest odds ratio is attained for the study of Nguyen et al.^24^ with 2.47. Since the majority of studies, i.e., Saseendram et al.^22^, Guimaraes et al.^14^, Guimaraes et al.^21^, and Kumm et al.^16^, consisted of patients in the age group 41–65 years, and resulted in an odds ratio above 1, i.e., 5.38, 51.68, 3.29, and 3.71 respectively, so it could be concluded that problems of meniscal tears increase with age.

**Table 3:** Age-related odds ratios for patient morbidity.

Patient’s age (years)	Publication	Odds ratio (95% CI)
02–61	Guimaraes et al. ^14^	51.68 (20.26–131.80)
41–61	Saseendram et al. ^22^	5.38 (1.580–infinite)
49–61	Englund et al. ^17^	74.3 (68.2–76.3)
17–19	Sproul et al. ^20^	3.58 (1.92–3.86)
31–71	Hagino et al. ^19^	4.662 (1.466–14.824)
43–65	Nguyen et al. ^24^	2.47 (1.64–3.94)
51–62	Guimaraes et al. ^21^	3.29 (1.28–8.44)
45–55	Kumm et al. ^16^	3.71 (0.98–6.03)

### Synthesis of results

**Figure 5:**
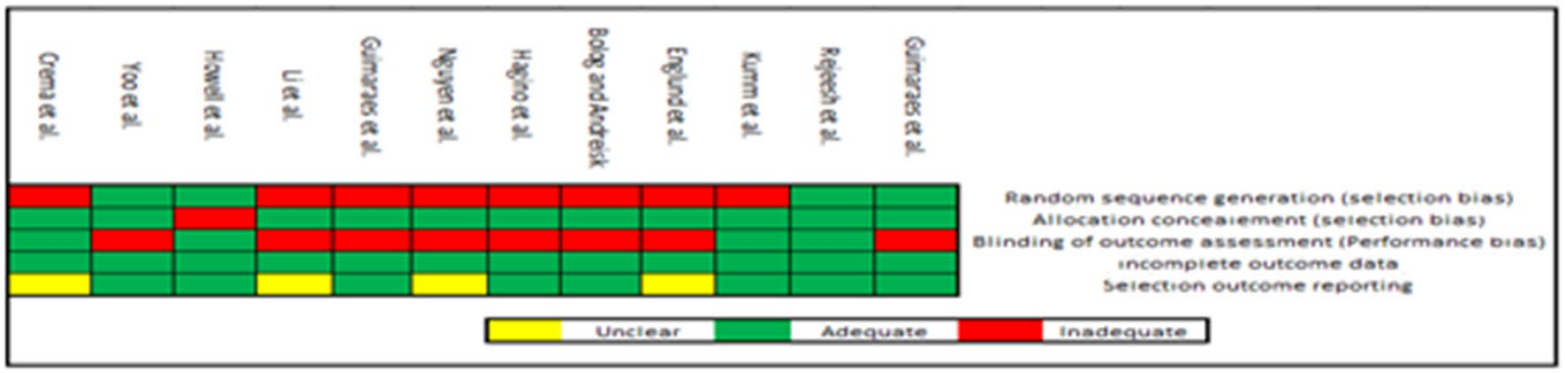
Odds of meniscal changes among patients.

The results are based on primary analysis of meniscal change in individual trials ranging between 3 and 24 months. The evaluation exhibits a small but statistically significant advantage for interventions, such as knee arthroscopy, compared to controls. At different postoperative time points, assessment between group differences provided a statistically significant advantage with respect to interventions, such as meniscal contusion at 3 months and 6 months. The results of the meta-analysis and forest plot are shown in Fig. 5. Pooled analysis for significant differences was notified (SMD 0.02, 95% CI − 0.31 to 0.45) and had low heterogeneity (P = 0.81, I^2^ = 0%). Similarly, significant differences have been notified for changes in meniscal contusion at 6 months (SMD 0.12, 95% CI 0.00–0.29).

## Discussion

The identification of intrasubstance meniscus change has been examined in a number of studies, owing to its significance and prevalence. A variety of findings have been identified in this review. Majority of the studies were prospective or retrospective cohorts and none among them was RCT. The results show that most studies report significant meniscal change with respect to age group and it could be summarized that problems of meniscal tears increase with age. The meta-analysis showed significant changes in meniscal contusion at 6 months. Weight control was found to be a strong preventive factor against developing true meniscal tears.

A study conducted by Guimaraes et al.^21^ investigated the relationship between the progression of meniscal intrasubstance degeneration and weight change in patients. Weight-gain is associated with an increased likelihood of meniscal intrasubstance degeneration progression. The degeneration was evaluated using the whole-organ magnetic resonance imaging score (WORMS) grading system, which is based on the following 14 features: posterior and anterior cruciate ligament integrity, articular cartilage integrity, sub-articular cysts, sub-articular bone attrition, sub-articular bone marrow abnormality, intraarticular loose bodies, synovitis/effusion, sub-articular bone marrow abnormality, periarticular cysts/bursitis, marginal osteophytes, medial and lateral meniscal integrity, medial and lateral collateral ligament integrity, cartilage signal and morphology, and loose bodies and periarticular cysts/bursae^26^. According to the WORMS grading system, meniscal changes are graded from grade 0 to 4, where 0 indicates normal meniscus, 1 refers to intrasubstance abnormalities, 2 indicates non-displaced tears, 3 refers to displaced or complex tearing, and 4 indicates complete maceration or complete resection of the meniscus. The study further added that grade 1 represents an unusual increase in intrasubstance signal on water-sensitive sequences, in which fluid is unreachable on the surface of the meniscus. While progression to true meniscal tear (WORMS ≥ 2) within 48 months signifies gradual change that occurred in patients with intrasubstance changes. The study also showed that meniscal tears were mostly developed because of excessive gain in weight within a period of 48 months, in contrast to normal weight gain. The conclusion of the study was that the increase in weight is directly related to the increase in meniscal degeneration; 60% of the overall subjects had undergone a progression of the problem owing to considerable weight gain. Also, a significant fivefold increased risk to develop a tear was observed in people with normal weight, whereas in patients with severe weight gain, a tenfold increase was observed in the progression of meniscal intrasubstance change into true tears. Participants that exercised and were able to lose weight within the duration of 48 months showed a protective effect of the articular knee cartilage^27^. Yoo et al.^18^ add through their study findings that paediatric patients with meniscal changes of grade 1 and 2 cannot accurately be predicted to have a true tear.

Saseendram et al.^22^ outlined the presence of linear intrameniscal signal intensity on MRI as a major risk factor for medial degenerative meniscal tear in the future. The meniscal injury of grades 1 and 2 can be presented with swelling, pain, and other mechanical symptoms, although most patients are actually asymptomatic^28^. Some patients identified other symptoms, including clicking or locking knees^29^. It is important to understand the micro-anatomic gross-anatomic features of the meniscus before selecting treatment in order to maintain the meniscal function. Also, a proper and in-depth understanding of meniscal tears and intrameniscal change is required to provide accurate treatments^30^.

Low et al.^23^ conducted a study to identify the clinical significance of intrasubstance meniscal lesions in MRI. According to the study, only 10% of patients were identified with grade 2 meniscal changes with a slight representation of meniscal tears. To clarify this further, a pilot study was conducted with ten symptomatic patients with a mean age of 28 years. A grade 2 meniscal signal on MRI was detected. Following the findings of arthroscopy, intrasubstance tear was found among all the included patients. Partial meniscectomy was also performed with a mean follow up of 6.7 months. The study thus concluded that grade 2 meniscal changes should be reported in a manner where clear signs of intrasubstance changes are observed. However, in cases where intrameniscal changes remain unclear, arthroscopy must be performed for symptomatic patients as this could improve the symptoms dramatically.

Menisci, on account of their internal structure, work as shock stabilizers. However, any change in the meniscus contributes to the development of stress on the various anatomical knee structures^31^. For accurate functioning, menisci require good biomechanical integrity and stability^28^. Crema et al.^15^ found a significant association between cartilage loss in the presence of true surfacing meniscal tears, while with only intrasubstance change, no significant increase in cartilage loss was observed. This indicates that intrasubstance change does not affect meniscus function unless a tear has formed. Wirth et al.^32^ add to this that the development of horizontal meniscus tears is generally associated with meniscal cysts, and can be detected during MRI. A high signal collection is best demonstrated in the coronal weighted images indicating the presence of horizontal tear. Nguyen et al. ^24^ supported this way of differentiating horizontal tear from intrasubstance changes, and added a more detailed radiological approach using “two-slice-touch” rule in which a true tear must be seen in two consecutive images at one plane or in one image at two different planes. Additionally, they add other associated findings that support the presence of a tear, such as meniscal extrusion. Intrasubstance changes, according to Nguyen et al. ^23^ are not at risk of developing tears in the future. Meniscal tears usually occur in association with degenerative knee joint disease or ligamentous injury, especially of the ACL^24,33^. Also, these studies found that most of the intrasubstance changes were observed among young adults, while in children the changes were found to be created by the meniscal vessels.

Guimaraes et al. ^21^ found that MRI findings of intra-meniscal changes are commonly seen in patients with ACL tears, however, the changes showed a benign course over a follow-up period of 24 months with no further development into meniscal tears. In addition, no significant difference was found in knee injury and osteoarthritis outcome scores (KOOSs) between the initial stage and after 24 months between subjects with and without intrameniscal signal intensity alteration. Magnussen et al. ^34^ studied the development of osteoarthritis that took place post ACL reconstruction, which is an important morbidity among young people. The findings of that systematic review concluded that the risk of developing osteoarthritis increased among patients that have undergone a meniscectomy at the time of ACL reconstruction. From this, we can conclude that surgically treating intrameniscal change may have a harmful long-term effect, even if there is an associated high risk, such as an ACL tear. By contrast, Howell et al. ^25^ identified a number of different treatment options for patients with symptomatic intrasubstance meniscal changes, which include surgical and non-surgical interventions. Both options showed effectiveness in the short term, but further research is recommended to better understand the long-term clinical effect.

No radiographic changes were observed in 78% of patients, whereas repairing of degenerative changes were observed among 34% patients, from grade 1 and grade 2 categories^35^. Kumm et al. ^16^ found that the linear intrameniscal signal intensity in MRI in middle-aged individuals is not possible to be resolved over a 6-year follow-up. The presence of such findings is a significant risk factor for medical degenerative meniscal tear^36^.

Patients with an unclear cause of knee pain generally undergo MRI in order to detect meniscal tears based on the attributed symptoms. For this purpose, Englund et al. ^17^ conducted a study on the prevalence of incidental findings of intrasubstance change across various age groups (middle-aged and elderly), including 991 patients. The prevalence rate of meniscal tears detected by MRI was about 19% among women and 32% among men aged 50–59 years. The incidence was much higher in older patients, with 51% in females and 56% in males aged 70–90 years. A higher prevalence rate for meniscal destructions was found in females, especially in the older age groups. In all patients with grade 1 and grade 2 meniscal changes, there were no visible signs of meniscal tears, even when there is excessive knee pain and stiffness. In fact, patients with grade 1 meniscal change usually had collateral damage when treated with bioabsorbable fixation device, which is used in repairing the meniscal tears^34^.

In certain cases, it is difficult to detect meniscal change if the high signal intensity of MRI is limited to the meniscus, or, it is extended to the articular surface. A similar idea was examined in Sproule et al. ^20^, who investigated the occurrence of borderline findings in high signal intensity MRI in the posterior horn of meniscal tears. Sixty-four patients were retrospectively investigated that were suspected of post-traumatic internal derangement of the knee. Patients also had MRI prior to arthroscopy. Ten patients were diagnosed with grade 2 changes equivocally. Also, the findings regarding the correlation of arthroscopic results with MRI indicated a single tear in the unequivocal group. The study therefore concluded that the use of MRI must be carefully considered when detecting meniscal change. It is further recommended to consider clinical history and examine for internal derangement of the knee.

According to Table 2, no significant difference was identified between the participant age group and meniscal change. Most participants consisted of a mean age of 35 years, with P-value above 0.001, which indicates that adult patients were identified with significant meniscal change. It can be further posited that meniscal changes are more common in older patients, but the development of true meniscal tear does not significantly increase with age. However, Table 3 provide findings in relation to age-related odds for patients’ morbidity: the highest odds ratio was observed for the study of Englund et al.^17^, with patients of 49–61 years, with an odds ratio of 74.3. The smallest odds ratio of 2.47 was observed for Nguyen et al. ^24^, which consisted of patients in the age group of 43–65 years. Other included studies, such as Guimaraes et al.^14^, Kumm et al. ^16^, and Saseendram et al. ^22^, ranged between 3.29 and 5.38. Following these findings, it may be concluded that the occurrence of meniscal tears among patients is generally independent of age.

The accurate diagnosis of meniscal change is based on criteria such as sequence parameters and experience and knowledge of meniscal anatomy^30^. Patients with intrasubstance meniscal change are usually asymptomatic and will often not require surgical intervention. The present study results help identify clinical correlations of grade 1 and grade 2 meniscal changes, which need to be differentiated from true intrasubstance horizontal tear. The intrameniscal changes usually follow a benign course in relation to various presenting symptoms and clinical outcomes. However, the presence of intrasubstance meniscal changes is considered a significant risk factor for developing a true tear later in life, which was often observed if patients gain weight. There were no significant increased risk factors found for other situations, even when it was accompanied by an ACL tear. The present study has highlighted the importance of reporting intrasubstance meniscal change when patients suffer from mechanical meniscal symptoms. It is important to report these changes with asymptomatic older patients, because this condition can develop into true tears, especially with increasing weight or the presence of a coexisting ligamentous tear.

The study has been limited as it included only studies published which might have caused publication bias. Furthermore, studies only in English were included, therefore there might have been language bias as well. Only a limited number of studies were included in the study and they were limited in reporting patient reported outcomes.

## Conclusion

The present study has evaluated the rate of encountered grade 1 and grade 2 intrasubstance meniscal changes toward guidelines for reporting alterations appropriate to predicted clinical outcomes. Various important relationships and correlations between the progression of meniscal intrasubstance degeneration and observed clinical symptoms, physical conditions of patients, and supporting MRI results, were derived, and can aid distinguishing intrasubstance changes from true tear. For the majority of patients, undergoing meniscal change remains asymptomatic and requires follow-up only when patients become symptomatic. However, surgically treated symptomatic patients are at risk of chondral damage. Weight control was found to be a strong preventive factor against developing true meniscal tears.
